# DSM‐5 Functional Framework: Structure and Psychopathological Correlates of the DSM‐5 Levels of Functioning Questionnaire–Short Form

**DOI:** 10.1002/pmh.70056

**Published:** 2025-12-26

**Authors:** Claudio Sica, Corrado Caudek, Ilaria Colpizzi, Gioia Bottesi

**Affiliations:** ^1^ Department of Health Science University of Florence Florence Italy; ^2^ Department of Neurosciences, Psychology, Drug Research, and Child Health University of Florence Florence Italy; ^3^ Department of Life Sciences University of Trieste Trieste Italy; ^4^ Department of General Psychology University of Padua Padua Italy

**Keywords:** alternative model for personality disorders, DLOPFQ‐SF, HiTOP, Level of Personality Functioning, psychometric validity

## Abstract

Interest in the Level of Personality Functioning (LPF), defined by Criterion A of the Alternative Model for Personality Disorders (AMPD), is growing. LPF includes self‐functioning (identity, self‐direction) and interpersonal functioning (empathy, intimacy). While LPF is crucial for understanding personality dysfunction, its ability to distinguish personality disorders from other conditions remains debated. The DSM‐5 Levels of Functioning Questionnaire–Short Form (DLOPFQ‐SF) is a promising LPF measure, but further research in diverse populations is needed. This study examined the internal structure and psychopathological correlates of the DLOPFQ‐SF in an Italian nonclinical sample. Specifically, it assessed the tool's factor structure and relations with the Hierarchical Taxonomy of Psychopathology (HiTOP) and positive adjustment. A sample of 1183 Italian participants (46.2% female, mean age = 31.3 ± 14.8) completed the DLOPFQ‐SF and other self‐report measures. Exploratory and confirmatory factor analyses showed a three‐factor structure comprising Identity, Empathy, and Intimacy. Structural equation modeling (SEM) explored links between the three DLOPFQ‐SF dimensions, HiTOP dimensions (internalizing, externalizing‐antisocial, antagonism, detachment, psychoticism), and positive adjustment. In general, SEM analyses showed that higher LPF scores were associated with increased psychopathology and reduced well‐being. The three subscales demonstrated specific links to distinct psychopathological outcomes, thereby supporting the construct validity of the DLOPFQ‐SF. Despite the removal of the Self‐Direction scale, the instrument proved useful in capturing key aspects of LPF across self and interpersonal domains, reinforcing its relevance within a dimensional model of psychopathology.

## 1 Introduction

Developments in the conceptualization and evaluation of personality disorders (PDs) emphasize a dimensional approach, as reflected in the inclusion of the ‘Alternative Model for Personality Disorders’ (AMPD) in Section III of the DSM‐5‐TR (*Diagnostic and Statistical Manual of Mental Disorders*, 5th Edition, Text Revision) (American Psychiatric Association 2022). This framework represents a significant shift from traditional categorical methods, offering a dimensional viewpoint on PDs that integrates impairments in *personality functioning* (Criterion A) with *maladaptive personality traits* (Criterion B).

Criterion A describes difficulties in self‐functioning, including identity and self‐direction, as well as challenges in interpersonal relationships, such as empathy and intimacy. This conceptualization builds on diverse psychological traditions, including psychodynamic, attachment‐based, and interpersonal theories (Mulay et al. 2018). Deficits captured by Criterion A are encapsulated in the construct of the Level of Personality Functioning (LPF) (Bender et al. 2014), which is considered a core indicator of the severity of personality dysfunction. By assessing impairments in these domains, the LPF framework provides a means of differentiating PDs from other psychological conditions. Indeed, many theoretical models of PDs focus on identity dysfunction (Kernberg 2006; Sharp and Wall 2018) and interpersonal pathology (Luyten and Blatt 2013). The LPF is therefore used to evaluate the severity of personality dysfunction and to help detect the presence of PDs.

While Criterion A plays a central role in identifying PDs, ongoing debates question its necessity and relation to Criterion B. Some researchers argue that Criterion A is essential to distinguish PDs from other disorders (Morey et al. 2022), while others contend that it overlaps significantly with Criterion B and offers limited diagnostic utility. Clearly, further research is necessary to shed light on this issue (Zimmermann 2022).

### 1.1 The DSM‐5 Levels of Functioning Questionnaire–Short Form

The LPF construct can be assessed through various methods, including structured clinical interviews (Bender et al. 2018) and self‐report questionnaires, which are valued for their efficiency and accessibility (Waugh et al. 2021). Self‐report instruments have generally been shown to capture the two fundamental dimensions of personality functioning—self and interpersonal functioning—providing valuable insights into personality pathology (Hopwood 2024).

Among these self‐report tools, the DSM‐5 Levels of Functioning Questionnaire–Short Form (DLOPFQ‐SF) has emerged as a promising option. Despite its brevity, initial studies (Siefert et al. 2020) indicated that DLOPFQ‐SF retains the strong psychometric properties of its longer counterpart, that is, the DLOPFQ (Huprich et al. 2018). Research on the full‐length measure demonstrated that the pool of items was internally consistent, with individual items grouping into four distinct subscales that align with key aspects of LPF: identity, self‐direction, empathy and intimacy. A second‐order factor encompassing these subscales sustained the use of a total score as a summary index of overall LPF (Huprich et al. 2018). Furthermore, similar to prior research (Dowgwillo et al. 2018), scores derived from the DLOPFQ were related to various personality dysfunction indicators, including traits such as overdependence, detachment, healthy dependency, and insecure attachment. Interestingly, associations between the total score and these indicators were generally stronger than those observed for specific subscales, highlighting the utility of the global LPF measure (Huprich et al. 2018).

Confirmatory factor analysis (CFA) has provided consistent evidence for the structural validity of the DLOPFQ‐SF. Findings indicated strong correlations between its four subscales and those of the full‐length measure, as well as significant relations with theoretically relevant constructs (Siefert et al. 2020). However, further research is required to explore its psychometric robustness across diverse samples, particularly outside of the United States, and to examine whether its four subscales offer unique predictive value for specific psychopathological outcomes: In fact, limited attention has been given to the relations between individual LPF dimensions and specific forms of psychopathology. Investigating these connections could offer more detailed insights into how core aspects of personality functioning contribute to the expression of various disorders.

### 1.2 The Current Study

This study seeks to contribute to the existing body of research by exploring the structure and psychopathological correlates of the DLOPFQ‐SF in an Italian nonclinical sample.

First, we assessed the internal structure of the DLOPFQ‐SF using a CFA approach. We hypothesized that the internal structure would include an overarching factor representing overall LPF, along with either two or four sub‐dimensions aligned with theoretical models of self and interpersonal functioning (Hopwood 2024; Siefert et al. 2020; Huprich et al. 2018).

Second, we evaluated the external validity of the DLOPFQ‐SF through structural equation modeling (SEM). This analysis aimed to examine how the questionnaire scores relate to key dimensions of the Hierarchical Taxonomy of Psychopathology (HiTOP) model, a comprehensive, dimensional framework for categorizing psychopathological phenomena (Kotov et al. 2017). HiTOP organizes psychological conditions hierarchically, with broader psychopathological spectra at higher levels and more specific symptoms and traits at lower levels (Cicero et al. 2024). Our study specifically targeted five dimensions of psychopathology: internalizing, two variants of externalizing (antisocial externalizing and antagonistic externalizing), detachment, and psychoticism. To date, the relations between LPF as assessed by the DLOPFQ‐SF and HiTOP dimensions remain unexplored. However, research has demonstrated that deficits in personality functioning are linked to a broad spectrum of psychopathological symptoms (Kerber et al. 2024). Based on this evidence, we anticipated that overall LPF scores would exhibit positive correlations with all broad HiTOP dimensions. Moreover, we expected self‐functioning domains to show stronger associations with internalizing symptoms and psychoticism, given the prominence of distress and self‐doubt in these conditions. We also hypothesized that interpersonal functioning would be closely related to antisocial and antagonistic behaviors, as these behaviors are particularly reflective of the interpersonal deficits characteristic of PDs (Wright et al. 2022). Nonetheless, due to the scarcity of studies directly addressing these issues, we refrained from formulating detailed predictions regarding specific LPF dimensions.

Third, we investigated the relation between the DLOPFQ‐SF and positive adjustment, considering the scarcity of studies focused on Criterion A and positive adaptation. Since personality functioning can be viewed as a spectrum ranging from adaptive to maladaptive, encompassing the intertwined abilities to understand oneself and relate to others, we expected that it would exhibit a negative correlation with positive adjustment (Łakuta et al. 2023).

## 2 Methods

### 2.1 Participants and Procedure

An initial sample of 1678 Italian nonclinical individuals, with 42.3% being undergraduate students, entered the study. Participants completed an extensive online survey on personality characteristics, which included measures in three main areas: adaptive and maladaptive personality traits, clinical symptoms and positive adjustment.

Some participants (*N* = 284) completed only a small part of the survey and were therefore excluded from the analysis. To ensure data quality for the remaining participants, their responses were assessed using the Triarchic Assessment Procedure for Inconsistent Responding (TAPIR) (Mowle et al. 2017), a validity index based on the Triarchic Psychopathy Measure (TriPM). This process identified 211 participants who provided inconsistent responses to the TriPM, and they were also excluded. As a result of these exclusions, the final dataset comprised 1183 participants.

Participants were briefed on the aims of the study and provided informed consent before participation. The research is part of a broader project and was approved by the Ethics Committee of Psychological Sciences at the University of Padova (Italy), adhering to the principles of the Declaration of Helsinki.

### 2.2 Measures

A detailed description of each measure used in the current study, along with references, is reported in the Supporting Information.

#### 2.2.1 The DSM‐5 Levels of Functioning Questionnaire‐Short Form

The DLOPFQ‐SF (Siefert et al. 2020) is a 24‐item questionnaire requiring respondents to rate items on a scale ranging from 1 (*strongly disagree*) to 6 (*strongly agree*). Items evaluate the four dimensions of the AMPD Criterion A across social relationships and work/school. The two “self‐domains” are Identity and Self‐Direction, and the two “other‐domains” are Empathy and Intimacy. Respondents assess each item based on their functioning in personal relationships and then reassess the same items in the context of work (or school). This results in the calculation of four scales: Identity, Self‐Direction, Empathy, and Intimacy. Each scale also includes subscale scores for Social Relationships and Work/School. Higher scores reflect greater levels of pathology. The translation process of the Italian DLOPFQ‐SF followed standard psychology guidelines (Brislin 1986).

#### 2.2.2 Internalizing Symptomatology

We measured internalizing symptoms through the scales of Depression and Anxiety of the Depression Anxiety Stress Scales‐21 (DASS‐21).

#### 2.2.3 Externalizing Symptomatology

To evaluate externalizing symptoms, we used the Self‐Report Delinquency Scale (SRDR), measuring engagement on a delinquent activities' checklist over the past 6 months, in particular, through the scales of Cheating/Rules and Damage.

#### 2.2.4 Antagonism, Detachment, and Psychoticism Symptomatology

We used the facet scores composing the Antagonism, Detachment, and Psychoticism domains of the Personality Inventory for DSM‐5 Personality Disorders (PID‐5).

#### 2.2.5 Positive Adjustment

To measure positive adjustment, we administered the P scale of the Scale of Positive and Negative Experience (SPANE‐P), assessing positive affect; the Flourishing Scale (FS), which evaluates the eudaimonic aspects of well‐being; and the Rosenberg Self Esteem Scale (RSES), which provides a measure of self‐esteem.

### 2.3 Statistical Analysis

Exploratory factor analyses (EFAs) were conducted in the R statistical computing environment (version 4.2.2) using the *psych* package, with *GPA rotation* for factor rotation and *polycor* for the computation of polychoric correlation matrices. Two extraction methods were employed to ensure the robustness of results: maximum likelihood estimation for continuous data and principal axis factoring applied to polychoric correlations for ordinal data. In both cases, an oblimin rotation was used to allow for correlated factors.

CFA and SEM models were computed through the CFA and SEM procedures available in the *lavaan* package. To evaluate the model fit, we considered the *χ* ^2^ test statistic, the comparative fit index (CFI), the root mean square error of approximation (RMSEA), and the standardized root mean square residual (SRMR). Given that the *χ* ^2^ test is highly sensitive to sample size, CFI values of 0.90 or higher are interpreted as indicating an acceptable fit (Hox et al. 2010). Similarly, RMSEA values below 0.05 reflect a close fit, while values between 0.05 and 0.08 are considered acceptable. For SRMR, values range from 0 to 1.0, with well‐fitting models typically scoring below 0.05, although values up to 0.08 are still acceptable^1^ (Hu and Bentler 1999).

Lastly, internal consistency and reliability of the identified factors were evaluated by computing Cronbach's *α* and McDonald's *ω* using the *psych* package.

The minimum sample size was calculated before data collection based on typical effect sizes in personality‐behavior studies (Funder and Ozer 2019) that reported an explained variance (*r* ^2^) of around 0.04. For a power of 0.8, a minimum of 347 participants was required. To enhance robustness, we aimed to exceed this number in recruitment.

## 3 Results

### 3.1 Factor Structure of the DLOPFQ‐SF

The factorial validity of the instrument was examined through a sequential analytic strategy combining confirmatory and exploratory approaches. Complete results for the analyses described in this section are documented in the code file uploaded on OSF.

We began with a series of CFAs testing theoretically plausible structures (one‐factor, two‐factor, four‐factor, higher order, and bifactor models), both with and without item collapsing to control for method variance. Across all specifications, model fit remained unsatisfactory (e.g., one‐factor solution: CFI = 0.37, RMSEA = 0.135; bifactor solution: CFI = 0.55, RMSEA = 0.117; four‐factor correlated solution with collapsed items: CFI = 0.79, RMSEA = 0.085). These results indicated that none of the initially hypothesized CFA structures adequately represented the data. Furthermore, the negligible method variance (about 5%) provided justification for collapsing the items.

Given these findings, we turned to EFA to reassess the dimensionality of the instrument. Initial EFAs were conducted specifying a four‐factor solution to evaluate the theoretical structure. Two analytic approaches were employed to ensure robustness: maximum likelihood estimation with continuous data and principal axis factoring with polychoric correlations to account for the ordinal nature of Likert‐scale responses. This dual approach provided convergent evidence across estimation methods.

Examination of the four‐factor solutions revealed substantial psychometric concerns regarding the Self‐Direction scale across both methods. In both analytic approaches, the Self‐Direction items showed unstable and problematic loading patterns. As a consequence, the Self‐Direction scale was excluded from subsequent analyses. This decision was guided by established psychometric principles emphasizing measurement quality and construct validity over theoretical completeness (Worthington and Whittaker 2006).

Following the exclusion of the Self‐Direction scale, we conducted EFAs on the remaining 15 items specifying a three‐factor solution. When comparing the models based on continuous versus ordinal data, the continuous‐data solution was preferred, as it offered clearer conceptual distinctions among factors, the absence of negative loadings, and a substantially higher proportion of explained variance (36% vs. 20%). Accordingly, we retained the continuous‐data model and carried forward only those items with factor loadings above 0.30.

Subsequent CFAs then tested three competing specifications of this three‐factor structure: correlated three‐factor, higher order, and bifactor models. Across these models, the correlated three‐factor and higher order models showed good fit indices (CFI = 0.91, RMSEA = 0.07, SRMR = 0.05, for both models). The non‐hierarchical model showed superior numerical stability during estimation procedures, while attempts to fit complex hierarchical structures (i.e., bifactor model) encountered convergence difficulties. Moreover, the somewhat moderate magnitude of inter‐factor correlations (on average 0.48) suggested that while the three factors share some common variance, this overlap does not necessitate the specification of a higher order general factor: the correlated factors model adequately captures these relations through direct covariances without introducing the additional complexity inherent in hierarchical structures.

Thus, from a practical standpoint, the equivalent predictive validity made the correlated 3‐factor solution the most appropriate choice for subsequent analyses. This decision aligns with established principles of model selection, where equivalent‐performing models favor the more parsimonious alternative that maintains theoretical coherence while ensuring empirical tractability.

In this solution, items all loaded onto respective factors at > 0.50 but two (Item 52 = 0.36; Item 43 = 0.41; see Table 1). The three factors exhibited on average a moderately strong correlation with each other (0.52), the lowest being 0.39 (Identity with Empathy) and the highest 0.62 (Identity with Intimacy).

**TABLE 1:** Factor loadings for the three factors of the DSM‐5 Levels of Functioning Questionnaire‐Short Form.

Item	Identity	Empathy	Intimacy
6	0.65		
7	0.63		
9	0.82		
10	0.81		
12		0.53	
15	0.56		
25	0.68		
32		0.63	
41		0.62	
43		0.41	
48		0.72	
49			0.68
51			0.76
52			0.36
54			0.65

*Note:* All items are significant at *p* < 0.001. Consistent with Siefert et al. (2020), items are identified according to the numeration used in the original full‐length DLOPFQ (Huprich et al. 2018).

Finally, the three scales obtained by summing the items of each factor showed satisfactory internal consistency. The Identity factor (6 items: Item 6, Item 7, Item 9, Item 10, Item 15, Item 25; M = 19.18, SD = 6.65) demonstrated good reliability, with Cronbach's *α* = 0.85 and McDonald's *ω* = 0.85. The Empathy factor (5 items: Item 12, Item 32, Item 41, Item 43, Item 48; M = 10.64, SD = 4.03) yielded acceptable reliability indices (*α* = 0.71, *ω* = 0.72). The Intimacy factor (4 items: Item 49, Item 51, Item 52, Item 54; M = 13.37, SD = 4.31) also showed acceptable reliability (*α* = 0.70, *ω* = 0.72).^2^

### 3.2 Associations Between LPF and HiTOP Dimensions

To index broader dimensions of internalizing, externalizing–antisocial, antagonism, detachment, and psychoticism psychopathology along with positive adjustment, we computed composites of specific criterion measures as outcome variables (detailed information is available in the Supporting Information). We then fitted two measurement models: In the first model, we specified five correlated HiTOP factors, while in the second model, we specified a hierarchical HiTOP model in which these five dimensions loaded onto a second‐order latent construct, representing general psychopathology (the “p factor”). Only the correlated five‐factor model converged with adequate fit, yielding CFI = 0.95, RMSEA = 0.08, and SRMR = 0.04 (factor loadings are reported in the Supporting Information, Table S1).

Lastly, SEM was used to investigate the relation between the DLOPFQ‐SF dimensions and broad outcome factors. To examine the joint structure of the LPF and the HiTOP models, we specified the three correlated LPF factors and the HiTOP measurement model, allowing the five HiTOP dimensions to correlate freely. We then estimated cross‐model correlations between the three LPF factors and each of the five HiTOP dimensions, thereby testing the extent to which latent personality traits were associated with psychopathology dimensions.

To avoid the statistical complications inherent in multiple comparison procedures, we employed a nested model comparison approach (Kline 2023). Specifically, we compared a null model, in which all cross‐domain correlations were constrained to zero, with a full model in which these correlations were freely estimated (yielding 15 cross‐domain parameters). The models were compared using the scaled chi‐square difference test appropriate for MLR estimation (Satorra and Bentler 2001), alongside changes in fit indices according to recommended thresholds (Chen 2007). A significant improvement in model fit for the full model would indicate that LPF and HiTOP domains are systematically intercorrelated, supporting theoretical expectations of convergence between personality pathology and psychopathology frameworks.

Model comparison indicated a highly significant improvement in fit for the full model over the null model, Δ*χ* ^2^(15) = 924.53, *p* < 0.001, demonstrating that allowing LPF and HiTOP domains to correlate significantly enhanced model adequacy. This finding supports the theoretical expectation of systematic interrelations between personality pathology and psychopathology dimensions. Practical significance was confirmed: ΔCFI = 0.073, ΔRMSEA = 0.017, and ΔSRMR = 0.112, all exceeding recommended thresholds (Chen 2007), indicating meaningful improvement in fit.

Examination of individual standardized cross‐domain correlations revealed robust associations between LPF and HiTOP factors (see Table 2). The Identity factor was positively associated with Internalizing (*ζ* = 0.683, *p* < 0.001), Detachment (*ζ* = 0.623, *p* < 0.001), and Psychoticism (*ζ* = 0.571, *p* < 0.001). The Empathy factor showed the strongest association with Detachment (*ζ* = 0.782, *p* < 0.001) and additional positive associations with Internalizing (*ζ* = 0.374, *p* < 0.001), Psychoticism (*ζ* = 0.354, *p* < 0.001), and Antagonism (*ζ* = 0.353, *p* < 0.001). The Intimacy factor was positively correlated with Detachment (*ζ* = 0.715, *p* < 0.001), Internalizing (*ζ* = 0.473, *p* < 0.001), and Psychoticism (*ζ* = 0.388, *p* < 0.001).

**TABLE 2:** Path coefficients for the SEM model predicting the six broad outcomes and the positive adjustment composite score from the DLOPFQ‐SF dimensions.

	Identity	Empathy	Intimacy
DLOPFQ‐SF → Internalizing	0.68	0.37	0.47
DLOPFQ‐SF → Externalizing Antisocial	0.27	0.20	0.20
DLOPFQ‐SF → Antagonism	0.25	0.35	0.15
DLOPFQ‐SF → Detachment	0.62	0.78	0.71
DLOPFQ‐SF → Psychoticism	0.57	0.35	0.39
DLOPFQ‐SF → Positive Adjustment	−0.82	−0.34	−0.51

*Note:* All correlations are significant at *p* < 0.001.

The same procedure was employed to evaluate the relations between the LPF dimensions and positive adjustment. Model comparison again demonstrated a highly significant improvement in fit for the full model relative to the null model, Δ*χ* ^2^(3) = 485.52, *p* < 0.001, indicating that the inclusion of cross‐domain correlations substantially enhanced model adequacy (ΔCFI = 0.100, ΔRMSEA = 0.030, and ΔSRMR = 0.116). Examination of individual standardized correlations revealed that the Identity factor showed a strong, negative, and highly significant association with Positive Adjustment (*r* = −0.820, *p* < 0.001), suggesting that higher levels of Identity were linked to lower adaptive functioning. Intimacy was also negatively associated with Positive Adjustment, with a moderate‐to‐strong effect size (*r* = −0.509, *p* < 0.001). Finally, Empathy exhibited a moderate negative correlation (*r* = −0.344, *p* < 0.001). Collectively, these results indicate that the LPF dimensions are robustly and consistently related to lower levels of Positive Adjustment, with Identity emerging as the most influential predictor.

## 4 Discussion

The current study aimed to examine both the internal structure of the DLOPFQ‐SF and the pathways to broad psychopathological outcomes modeled after HiTOP in a large nonclinical sample. Additionally, we explored the relations between LPF and positive adjustment. Overall, our findings were consistent with theoretical expectations, while the observed discrepancies offer valuable insights for further interpretation.

The internal structure of the DLOPFQ‐SF identified in our study was partially consistent with existing literature (Hopwood 2024; Siefert et al. 2020), revealing three well‐defined factors consistent with theory (i.e., Identity, Empathy, and Intimacy reasonably separated from each other) but not a higher order personality functioning factor.

The present findings reveal notable psychometric challenges specifically associated with the self‐direction domain of personality functioning assessment, which align with emerging concerns in the broader AMPD literature. For example, Kerr et al. (2023) examined the Levels of Personality Functioning Questionnaire 12–18 in an adolescent sample using a bifactor model with a general factor and four specific factors (i.e., Identity, Self‐Direction, Empathy, and Intimacy). Although the overall model fit was adequate, Self‐Direction contributed very little unique variance beyond the general factor, and psychometric indices suggested it was less well‐defined than the other subdomains. Again, Sleep et al. (2024) examined the structure of personality dysfunction by applying a bass‐ackward factor‐analytic approach to more than 250 items drawn from multiple LPF measures, including the DLOPFQ‐SF. Their results revealed four lower order factors—Negative Self‐Regard, Disagreeableness, Intimacy Problems, and Lack of Direction. While the measures generally showed strong associations with Negative Self‐Regard and Disagreeableness, correlations with Lack of Direction were lower, indicating that self‐direction may be a particularly challenging domain to assess reliably.

Additionally, the brief version of the DLOPFQ‐SF may have measurement limitations: Its self‐direction items emphasize submissiveness, which suggests that the self‐direction domain is only partially represented in the DLOPFQ‐SF. However, this challenge does not appear to be unique to the DLOPFQ‐SF. For instance, the widely used Level of Personality Functioning–Brief Form 2.0 (Weekers et al. 2019) demonstrates a two‐factor structure distinguishing Self and Interpersonal functioning. Notably, the scale includes almost no items explicitly targeting self‐direction (with the exception of a single item), indicating that this domain is minimally represented. Future research should focus on developing more refined methods for assessing self‐direction, potentially incorporating behavioral indicators or multi‐method approaches to better capture the complex and multifaceted nature of this domain of personality functioning. In the present context, the use of the DLOPFQ‐SF to assess LPF in Italian samples suggests that practitioners and scholars may need to supplement it with additional assessment methods to ensure a more reliable evaluation of self‐direction.

In this study, the absence of a second‐order factor likely reflects the centrality of Identity, which emerged as the strongest domain in terms of loading magnitude, internal reliability, and external validity. This observation is consistent with findings from content validity analyses of self‐report measures of LPF, which indicate that Identity tends to be prominently represented across different instruments (Waugh et al. 2021). Given that much of the common variance across domains is already captured by identity, a higher order construct becomes redundant, as empathy and intimacy primarily reflect residual variance once identity is accounted for. Moreover, it is possible that the absence of a second‐order factor in our analyses is partly attributable to the removal of the self‐direction scale. Within Criterion A, self‐functioning is conceptualized as comprising both identity and self‐direction. By excluding the latter, the balance of the model was altered, leaving identity as the sole robust indicator of self‐functioning. This likely amplified the dominance of identity while simultaneously reducing the unique variance that could have been captured by a higher order construct. At the same time, our findings may speak to a broader conceptual issue that has been debated in literature: whether the LPF should be regarded as a single dimension or as a set of partially independent components. While the DSM‐5 Personality and Personality Disorders Work Group originally described LPF as a unitary construct, more recent contributions suggest that it may be better understood as multidimensional, with distinct domains that display different patterns of external associations (Sleep et al. 2024; Sleep et al. 2019). From this perspective, the prominence of identity and the lack of a higher order factor in our data may reflect not only methodological features of the present study but also the possibility that personality functioning is inherently multifaceted, and that its domains are best studied in relative independence rather than as indicators of a single continuum.

Findings on the psychopathological correlates of LPF also point in this direction: each of the DLOPFQ‐SF factors showed indeed some specificity. The identity domain was linked to all outcome criteria, underscoring the importance of a stable, coherent identity for emotional stability and psychological well‐being (as shown by the strong negative association between identity and positive adjustment). Again, the present findings align with theoretical models positioning identity disturbance as the fundamental organizing feature of personality pathology (Kernberg and Caligor 2005; Sharp and Wall 2021). Consistent with theory and prior research (Wright et al. 2022), Empathy showed strong associations with Detachment and moderate associations with Psychoticism and Antagonism. All these HiToP dimensions are in fact associated with deficits in empathy and interest towards others. Moreover, the coldness and lack of interest typical of low empathy were negatively linked to internalizing symptoms and had a weak negative association with positive adjustment. This suggests that individuals with low empathy may avoid or suppress their emotions, a pattern seen in antisocial or highly introverted personalities. These results support the value of this facet in assessing personality pathology.

Lastly, Intimacy occupies an intermediate position within the LPF framework: closely connected to Identity yet retaining distinctive features. The moderate–strong correlation between the two factors (*r* > 0.60) can be understood considering their shared developmental origins. From a developmental and attachment‐based perspective, the formation of a coherent identity and the capacity for intimacy are both rooted in early caregiver–child relationships. Secure attachment provides the infant with a stable sense of self‐continuity (“I am the same person across time and situations”) while simultaneously fostering trust in others as reliable and emotionally available. These parallel processes create the foundation for both self‐coherence (identity) and the later ability to establish reciprocal emotional bonds (intimacy) (Erikson 1963; Bowlby 1969). When early attachment experiences are inconsistent or disrupted, both domains may be undermined, producing the strong empirical linkage observed in our data. At the same time, Intimacy also demonstrates a distinctive profile. As part of the interpersonal domain of Criterion A, Intimacy showed particularly strong associations with Detachment, consistent with its role in capturing the ability to establish and sustain close, reciprocal relationships. Unlike Identity, Intimacy was only moderately related to Internalizing, Psychoticism, and Positive Adjustment. This suggests that while identity disturbance may permeate multiple life domains, intimacy dysfunction is somewhat more circumscribed in its effects on personality, reflecting specific vulnerabilities in close relational contexts. In this sense, identity and intimacy can be best viewed as interconnected but not interchangeable. Both emerge from similar developmental mechanisms, but they manifest differently across adulthood: identity as a pervasive marker of self‐structure and global impairment, intimacy as a relationship‐specific capacity that may remain selectively vulnerable even in the presence of a relatively intact sense of self (Erikson 1963; Bowlby 1969). Their overlap seems to underscore the centrality of self–other integration in Criterion A, whereas their divergence highlights the importance of assessing both domains when evaluating the nuances of personality pathology. Lastly, our finding that all LPF factors are linked to lower levels of positive adjustment further validates AMPD Criterion A, supporting the notion that LPF has clinical significance even in individuals without a formal PD diagnosis (American Psychiatric Association 2022).

Overall, our results align with prior research, showing that self‐report measures of LPF capture impairments relevant across mental disorders (Kerber et al. 2024) and reinforcing the original AMPD assertion that assessing personality‐related problems may be pertinent across various patient groups (Skodol 2012). More in general, LPF scores explained the largest amount of variance in the internalizing and detachment spectra compared to other ones. This pattern suggests that impairments in personality functioning are most visible in domains linked to social withdrawal, emotional distance, and vulnerability to internalizing problems. This finding also connects with recent perspectives that consider personality disorders from an interpersonal standpoint (Wright et al. 2022).

In contrast, LPF factors showed smaller associations with Antagonism and Externalizing (*r*'s ≤ 0.35). Taken together, these results support the view that Criterion A provides its clearest signal in relation to internalizing vulnerabilities and detachment from others, while offering less explanatory power for externalizing behavior (Sica et al. 2024). One plausible explanation for the weak LPF–Externalizing associations may relate to the possibility that individuals with pronounced externalizing tendencies—particularly those with antisocial features—may exhibit PF levels so low that self‐report items become difficult to be answered accurately. This interpretation is consistent with our operationalization of Externalizing, which relied primarily on indicators of delinquency and antagonism. Accordingly, evidence from a recent observer‐based study showed that people with antisocial personality disorder displayed markedly low personality functioning levels, comparable to those observed in severe psychotic disorders (Wendt et al. 2025). Additionally, findings from forensic samples generally demonstrate elevated impairment in personality functioning, with some studies reporting minimal correspondence between observer‐ and self‐reported LPF in such populations (Flechsenhar et al. 2024; Hutsebaut et al. 2021), suggesting that self‐report measures may underestimate impairment among individuals with extreme externalizing pathology.

The weak associations between LPF and externalizing symptoms may also reflect a conceptual distinction between the two. LPF, as operationalized in the AMPD Criterion A, is conceptualized as a severity dimension of personality pathology (Bender et al. 2014). Differently, externalizing symptoms reflect a pattern of behavioral disinhibition and antagonism that is more proximal to overt actions and symptom content rather than the underlying structural deficits in personality functioning (Krueger and Hobbs 2020). Consequently, externalizing symptoms, such as aggression and impulsivity, may be more appropriately understood as manifestations of specific dispositional patterns (Criterion B) rather than consequences of generalized functioning deficits (Criterion A). Some empirical findings appear to corroborate this distinction. For example, studies testing the external validity of LPF measures showed stronger associations with internalizing phenomena (e.g., negative affectivity, interpersonal sensitivity) and only modest or inconsistent associations with externalizing domains such as antisocial behavior or substance use (Sica et al. 2024). These patterns suggest that LPF primarily indexes generalized impairment in self‐ and relational functioning, which may underlie—but is not reducible to—the behavioral dysregulation captured by externalizing spectra (Boone et al. 2025). Further research employing more precise and differentiated measures of LPF is needed to clarify the nature of these associations and the extent to which LPF contributes to externalizing psychopathology.

### 4.1 Limitations

We acknowledge that our findings must be cautionary interpreted due to several shortcomings. First, the study's cross‐sectional design precludes any conclusions about causality between LPF and the examined psychopathological outcomes. Only longitudinal research can more robustly clarify these associations. Indeed, cross‐sectional SEM cannot determine whether LPF impairments lead to psychopathology, whether psychopathology affects LPF, or whether both are influenced by shared underlying factors. Bi‐directional or cross‐lagged panel models provide more informative insights into these dynamics. Second, although self‐report measures enabled us to recruit a large, representative community sample, they may have inflated associations due to shared method variance. Future research could address this by incorporating multi‐method, performance‐based assessments (Rucker et al. 2024). Third, since our sample consisted of nonclinical participants, the findings may not fully generalize to clinical populations. Fourth, although the HiTOP model includes the spectra of internalizing, thought disorder, detachment, disinhibited externalizing, and antagonistic externalizing, the present study did not incorporate any indicators of maladaptive traits associated with the disinhibited externalizing spectrum. The absence of measures for this domain represents a limitation, as our analyses only partially cover the full range of HiTOP dimensions. Nevertheless, recent evidence suggests that antisocial externalizing shows some degree of overlap with the domain of disinhibition (Sica et al. 2024), which may partially mitigate this omission. Lastly, we focused on the spectra level within the HiTOP hierarchy, as spectra tend to remain more stable over time than individual symptoms, providing a reliable measure of psychopathology that minimizes the effects of temporary or situational factors (Wright and Simms 2015). This level of classification is also highly relevant to clinical practice.

### 4.2 Conclusions and Clinical Implications

In conclusion, this study supports the clinical value of the Italian DLOPFQ‐SF in assessing key domains of personality functioning and their links to broader psychopathology. Its strong associations with HiTOP constructs suggest that LPF assessment may help identify risks such as emotional dysregulation, social withdrawal, and impaired self‐concept, even outside clinical settings. The connection between LPF and positive adjustment further underscores its role in resilience and well‐being. Incorporating LPF assessments into routine evaluations could therefore enhance detection of personality‐related vulnerabilities across diagnoses. Nonetheless, given the limits of self‐report measures and the challenges of the self‐direction domain, future research should pursue multi‐method approaches, including interviews, to improve the accuracy and clinical utility of LPF assessment.

## Author Contributions

Conceptualization: Gioia Bottesi, Claudio Sica. Data curation: Corrado Caudek, Ilaria Colpizzi. Investigation: Gioia Bottesi, Corrado Caudek, Ilaria Colpizzi. Project administration: Gioia Bottesi, Claudio Sica. Supervision: Gioia Bottesi, Claudio Sica. Writing – original draft: Gioia Bottesi, Claudio Sica. Writing – review and editing: Gioia Bottesi, Claudio Sica. All authors read and approved the final manuscript.

## Funding

The authors have nothing to report.

## Ethics Statement

The study was approved by the Ethics Committee for Psychological Research of the University of Padua and was conducted in accordance with the Declaration of Helsinki.

## Consent

Written informed consent was obtained from all participants.

## Conflicts of Interest

The authors declare no conflicts of interest.

## Supporting information


**Data S1:** Supporting Information.

## Data Availability

All data are publicly accessible on the Open Science Framework and can be retrieved at https://osf.io/qyzxu/?view_only=2a1d1eb8cec64f6abd7fd0ba7dc5f5b4.

## Endnotes

^1^
We sincerely appreciated the reviewer's recommendation to consider the unbiased SRMR (uSRMR) index in assessing model fit. Although the unbiased SRMR represents a methodological advancement by correcting for the traditional SRMR's downward bias in models with low communalities or small sample sizes (Shi et al. 2018; Ximénez et al. 2022), it was not employed here because mounting evidence indicating that the uSRMR, in its current formulation, operates as an exceedingly conservative fit index. Empirical simulation studies demonstrate this starkly: Under common research conditions (e.g., moderate communalities), the uSRMR can reject over 60% of correctly specified models at its proposed cutoff, a rate far exceeding the nominal alpha level (Ximénez et al. 2022). In applied research, where models are inherently approximations of reality (Browne and Cudeck 1992; Preacher et al. 2007), sole reliance on an index with such high stringency risks invalidating substantively meaningful models that adequately account for the data structure. Future methodological work should focus on developing conditional guidelines for the uSRMR and integrating it into broader fit assessment frameworks that balance statistical sensitivity with substantive interpretability.

^2^
The final version of the Italian DLOPFQ‐SF can be retrieved from the Open Science Framework folder.
