# Supplementary material for: DSM‐5 Functional Framework: Structure and Psychopathological Correlates of the DSM‐5 Levels of Functioning Questionnaire–Short Form
Source: Personal Ment Health. 2025 Dec 26;20(1):e70056. doi: 10.1002/pmh.70056 (PMC12741785; doi:10.1002/pmh.70056)
Supplement: Supplementary file 1 — Data S1: Supporting Information. [file PMH-20-0-s001.docx]

**Supplemental material**

**Measures**

*The Depression Anxiety Stress Scales-21 (DASS-21).* The DASS-21 (Lovibond & Lovibond, 1995) is a 21-item questionnaire measuring depression, anxiety, and stress over the previous week. Participants rate items on a scale ranging from 0 (*did not apply to me at all*) to 3 (*applied to me very much*). Both the original English-language and Italian version (Bottesi et al., 2015) demonstrated good psychometric properties. In the current study, internal consistency values were excellent (depression scale: α= .91; anxiety scale: α= .88; stress scale: α= .89).

*The Self-Report Delinquency Scale (SRDR)***.** In the SRDS, individuals report their involvement (*Yes/No*) in a 36-item checklist of delinquent activities over the past 6 months. Of these, 29 items were adapted from the 56-item checklist by Curcio et al. (2015) to suit the Italian context, while seven additional items were included to better capture behaviors such as cheating and fighting. The 36 items employed in this study underwent a Principal Component Factor Analysis with Promax Rotation. Parallel analysis, Cattell’s scree test and the factor interpretability supported a four-factor solution which explained the 33% of the total variance: Disturbing/Fight (involvement in minor crimes, school problems, fight; 12 items, α=.79); Cheating/Rules (conning, take advantage of others, not respecting rules; 8 items, α=.64); Damage (damages and negative consequences for others; 7 item, α=.60); Crime (dealing drugs, weapon possession and use; 7 items, α=.50)^[[1]](#footnote-1)^.

*The Personality Inventory for DSM-5 Personality Disorders (PID-5).* The PID-5 (Krueger et al., 2012) is made up of 220 items rated on a scale ranging from 0 (*very false*) to 3 (*very true*) evaluating the AMPD Criterion B 25 maladaptive personality traits, which load onto 5 higher-order dimensions: Antagonism, Detachment, Disinhibition, Negative Affectivity and Psychoticism. The Italian version of the PID-5 demonstrated acceptable psychometric properties (Fossati et al., 2013; see also, Bottesi et al., 2024). Within the current study sample, we considered the facets pertaining to the Antagonism, Detachment, and Psychoticism domains. Internal consistency values were: Manipulativeness = .82, Deceitfulness = .86, Grandiosity = .83 (Antagonism); Withdrawal = .78, Anhedonia = .76, Intimacy Avoidance = .79 (Detachment); Eccentricity = .94, Perceptual Dysregulation = .85; Unusual Beliefs = .81 (Psychoticism).

*The Scale of Positive and Negative Experience (SPANE-P).* The SPANE (Diener et al., 2010) is a 12-item tool comprising two subscales: positive affect (SPANE-P; 6 items) and negative affect (SPANE-N; 6 items). These subscales measure affective experiences over the past 4 weeks. Participants indicate the frequency of each feeling on a scale from 1 (*very rarely* or *never*) to 5 (*very often* or *always*). The Italian adaptation of the SPANE (Giuntoli et al., 2017) demonstrated a better fit for a two-factor model compared to a one-factor model, supporting the distinction between the two components. Concurrent validity was supported. For the purposes of this study, we only used the SPANE-P subscale. Internal consistency in the current sample was excellent (α = .92).

*The Flourishing Scale (FS).* The FS (Diener et al., 2010) is made up of 8 items, rated from 1 (*strongly disagree*) to 7 (*strongly agree*), which focus on the eudaimonic aspects of well-being. Higher scores indicate that the individual perceives themselves as highly positive in functioning. The Italian version of the FS (Giuntoli et al., 2017) has proven to be a reliable and valid. In the current sample, internal consistency (α) for the total score was .88.

*The Rosenberg Self Esteem Scale (RSES).* The RSES (Rosenberg, 1965) is a 10-item questionnaire measuring global self-worth by evaluating both positive and negative self-perceptions. Responses are provided on a scale ranging from *strongly agree* to *strongly disagree*. Widely regarded as a reliable and valid tool for measuring self-esteem, its Italian version showed good psychometric properties equivalent to the original one (Prezza et al., 1997). In the current study Cronbach’s αfor total score was .91.

**Composites of outcome variables**

As a foundation for aggregating individual indicators into composites of outcome variables, we performed a CFA using:

- Internalizing, measured by symptoms of anxiety and depression.
- Externalizing–Antisocial, measured by rule-breaking and aggression.
- Antagonism, measured by deceitfulness, manipulativeness, and grandiosity.
- Detachment, measured by anhedonia, withdrawal, and intimacy avoidance.

Psychoticism, measured by eccentricity, perceptual dysregulation, and unusual beliefs.

The facet scores of the PID-5 as indicators of the broad Antagonism, Detachment, and Psychoticism included under each domain as per formal scoring procedures (APA, 2014)^[[2]](#footnote-2)^. Moreover, a unit-weighted composite of the SPANE-P, the FS, and the RSES scores was computed to represent the broader dimension of "Positive Adjustment" score.

The fit of the model was good: CFI= .98, RMSEA= .08, SRMR= .04. The factor loadings are showed in Table S1.

**Table S1.** Factor loadings of the composites representing broader dimensions of externalizing, internalizing, antagonism, detachment and psychoticism psychopathology.

|  | **Externalizing Antisocial** | **Internalizing** | **Antagonism** | **Detachment** | **Psychoticism** |
| --- | --- | --- | --- | --- | --- |
| SRDS Cheating/Rules | .90 |  |  |  |  |
| SRDS Damage | .45 |  |  |  |  |
| DASS-21Anxiety |  | .78 |  |  |  |
| DASS-21 Depression |  | .88 |  |  |  |
| PID-5 Manipulativeness |  |  | .86 |  |  |
| PID-5 Deceitfulness |  |  | .91 |  |  |
| PID-5 Grandiosity |  |  | .64 |  |  |
| PID-5 Withdrawal |  |  |  | .63 |  |
| PID-5 Anhedonia |  |  |  | .87 |  |
| PID-5 Intimacy Avoidance |  |  |  | .47 |  |
| PID-5 Eccentricity |  |  |  |  | .75 |
| PID-5 Perceptual Dysregulation |  |  |  |  | .90 |
| PID-5 Unusual Beliefs |  |  |  |  | .75 |

*Note:* SRDS = Self-Report Delinquency Scale; DASS-21 = Depression Anxiety Stress Scales-21;; PID-5 = Personality Inventory for DSM-5 Personality Disorders.

References

Bottesi, G., Caudek, C., Malerba, A., Caselli, G., Gallo, G., Melli, G., Marsigli, N., Offredi, A., & Sica, C. (2024). Agreement and discrepancies in patient–clinician reports of DSM-5-TR section III maladaptive personality traits: A study on a mixed outpatient sample. Personality Disorders: Theory, Research, and Treatment, 15(1), 94–99.  https://doi.org/10.1037/per0000639

Bottesi, G., Ghisi, M., Altoè, G., Conforti, E., Melli, G., & Sica, C. (2015). [The Italian version of the Depression Anxiety Stress Scales-21: Factor structure and psychometric properties on community and clinical samples](http://www.comppsychjournal.com/article/S0010-440X(15)00056-5/abstract). *Comprehensive Psychiatry*, 60, 170-181. https://doi.org/[10.1016/j.comppsych.2015.04.005](https://doi.org/10.1016/j.comppsych.2015.04.005)

Curcio, A. L., Mak, A. S., & Knott, V. E. (2015). The Australian Self‐report Delinquency Scale: A

revision. *Australian Journal of Psychology, 67*(3), 166-177. https://doi.org/10.1111/ajpy.12075

Diener, E., Wirtz, D., Tov, W., Kim-Prieto, C., Choi, D. W., Oishi, S., & Biswas-Diener, R. (2010). New well-being measures: Short scales to assess flourishing and positive and negative feelings. *Social Indicators Research, 97*, 143-156. https://doi.org/10.1007/s11205-009-9493-y

Fossati, A., Krueger, R. F., Markon, K. E., Borroni, S., & Maffei, C. (2013). Reliability and validity of the personality inventory for DSM-5 (PID-5): predicting DSM-IV personality disorders and psychopathy in community-dwelling Italian adults. *Assessment*, *20*(6), 689-708. <https://doi.org/10.1177/1073191113504984>

Huprich, S. K., Nelson, S. M., Meehan, K. B., Siefert, C. J., Haggerty, G., Sexton, J., Dauphin, V. B., Macaluso, M., Jackson, J., Zackula, R., & Baade, L. (2018). Introduction of the DSM-5 levels of Personality Functioning Questionnaire. *Personality Disorders: Theory, Research, and Treatment, 9(*6), 553–563. https://doi.org/10.1037/per0000264

Giuntoli, L., Ceccarini, F., Sica, C., & Caudek, C. (2017). Validation of the Italian versions of the

Flourishing Scale and of the Scale of Positive and Negative Experience. *Sage Open*, 7, 1-12. https://doi.org/10.1177/2158244016682293

Krueger, R. F., Derringer, J., Markon, K. E., Watson, D., & Skodol, A. E. (2012). Initial construction of a maladaptive personality trait model and inventory for DSM-5. *Psychological Medicine*, 42(9), 1879-1890. https://doi.org/10.1017/S0033291711002674

Krueger, R. F., Markon, K. E., Patrick, C. J., Benning, S. D., & Kramer, M. D. (2007). Linking antisocial behavior, substance use, and personality: An integrative quantitative model of the adult externalizing spectrum. *Journal of Abnormal Psychology, 116*(4), 645–666. [https://doi.org/10.1037/0021-843X.116.4.645](https://psycnet.apa.org/doi/10.1037/0021-843X.116.4.645)

Lovibond, P. F., & Lovibond, S. H. (1995). The structure of negative emotional states: Comparison of the Depression Anxiety Stress Scales (DASS) with the Beck Depression and Anxiety Inventories. *Behaviour Research and Therapy, 33*(3), 335–343. [https://doi.org/10.1016/0005-7967(94)00075-U](https://psycnet.apa.org/doi/10.1016/0005-7967(94)00075-U)

Meyer, T. J., Miller, M. L., Metzger, R. L., & Borkovec, T. D. (1990). Development and validation of the penn state worry questionnaire. *Behaviour Research and Therapy, 28*(6), 487-495. https://doi.org/10.1016/0005-7967(90)90135-6

Morani, S., Pricci, D., & Sanavio, E. (1999). Penn State Worry Questionnaire e Worry Domains

Questionnaire. Presentazione delle versioni italiane ed analisi della fedeltà. *Psicoterapia Cognitiva e Comportamentale,* 5, 195-209.

Patrick, C. J., Kramer, M. D., Krueger, R. F., & Markon, K. E. (2013). Optimizing efficiency of

psychopathology assessment through quantitative modeling: Development of a brief form of

the Externalizing Spectrum Inventory. *Psychological Assessment*, 25(4), 1332-1348. https://doi.org/[10.1037/a0034864](https://doi.org/10.1037/a0034864)

Prezza, M., Trombaccia, F. R., & Armento, L. (1997). La scala dell'autostima di Rosenberg: Traduzione e validazione Italiana. *Giunti Organizzazioni Speciali, 223,* 35–44

Rosenberg, M. (1965). *Society and the Adolescent Self-Image*. Princeton University Press, Princeton.

Siefert, C. J., Sexton, J., Meehan, K., Nelson, S., Haggerty, G., Dauphin, B., & Huprich, S. (2020). Development of a short form for the DSM–5 levels of personality functioning questionnaire. *Journal of Personality Assessment, 102*(4), 516-526. https://doi.org/10.1080/00223891.2019.1594842

1. Two items did not appreciably load in any factor: “Driving without license”; “Sneak into someone’s else house”. [↑](#footnote-ref-1)
2. The three most representative facet scores included under each domain are as follows: Manipulativeness, Deceitfulness, Grandiosity (Antagonism); Withdrawal, Anhedonia, Intimacy Avoidance (Detachment); Cognitive/Perceptual Dysregulation, Eccentricity, Unusual beliefs/experiences (Psychoticism). [↑](#footnote-ref-2)
